# Machine Learning Identification of Piezoelectric Properties

**DOI:** 10.3390/ma14092405

**Published:** 2021-05-05

**Authors:** Mariana del Castillo, Nicolás Pérez

**Affiliations:** Faculty of Engineering, University of the Republic (UdelaR), 11300 Montevideo, Uruguay; nico@fisica.edu.uy

**Keywords:** neural network, FEM optimization, piezoelectric parameters

## Abstract

The behavior of a piezoelectric element can be reproduced with high accuracy using numerical simulations. However, simulations are limited by knowledge of the parameters in the piezoelectric model. The identification of the piezoelectric model can be addressed using different techniques but is still a problem for manufacturers and end users. In this paper, we present the use of a machine learning approach to determine the parameters in the model. In this first work, the main sensitive parameters, c_11_, c_13_, c_33_, c_44_ and e_33_ were predicted using a neural network numerically trained by using finite element simulations. Close to one million simulations were performed by changing the value of the selected parameters by ±10% around the starting point. To train the network, the values of a PZT 27 piezoelectric ceramic with a diameter of 20 mm and thickness of 2 mm were used as the initial seed. The first results were very encouraging, and provided the original parameters with a difference of less than 0.6% in the worst case. The proposed approach is extremely fast after the training of the neural network. It is suitable for manufacturers or end users that work with the same material and a fixed number of geometries.

## 1. Introduction

Piezoelectric ceramics are used in several technological devices. In some applications, the performance of the ceramics is evaluated using numerical simulations [1,2]. One of the most commonly used techniques is the finite element method (FEM). This method allows the precise determination of the deformation, the dynamical behavior, and the pressure field produced by the vibration of the ceramic when used as a transducer. However, the accuracy of these measures is limited by knowledge of the material properties. Thus, it is necessary to use various techniques to determine the parameters for the piezoelectric constitutive equations. These parameters are needed in order to simulate the behavior and develop new applications.

In the case of piezoelectric ceramics with a defined polarization direction and parameters that are not frequency dependent, the material has ten independent parameters: five elastic, three piezoelectric, and two dielectric constants (c11, c12, c13, c33, c44, e31, e15, e33, ε11, ε33). In this work, the imaginary part of the parameter will remain so, and only the real part of the parameters is considered. In this case, the piezoelectric model is expressed using reduced Voigt notation. Voigt notation reduces the order of the tensors by using the present symmetry. The physical quantities are tensors but are represented and manipulated as vectors and matrices. Strain and stress tensors are reduced to six-component vectors and the elastic tensor is reduced to a six-by-six matrix [3]. Assuming a linear model, the piezoelectric constitutive equations relating the stress T and electric displacement D can be described using this reduced notation, as shown in Equation (1).

(1) $$\begin{array}{c} T_{n}=c_{nq}^{E}S_{q}-e_{np}E_{p}\\ D_{m}=\varepsilon_{mp}^{S}E_{p}+e_{mp}S_{q}\\ \left(n=1:6;\;q=1:6;\;m=1:3;\;p=1:3;\right) \end{array}$$

Here, c, ε and e represent the elastic, dielectric, and piezoelectric matrices, respectively. The mechanical strain S and the electric field E are the independent variables. Super index E means constant electric field, whereas super index S represents constant strain. All parameters in the model may be complex to introduce the energy losses [4]. The real part mainly determines the frequency of the resonant modes, whereas the imaginary part introduces the losses. The reduced matrices are presented in Equation (2), where the representation in the case of the 6mm symmetry is shown.

(2) $$c_{ij}^{E}=\left[\begin{array}{cccccc} c_{11}^{E} & c_{12}^{E} & c_{13}^{E} & 0 & 0 & 0\\ c_{12}^{E} & c_{11}^{E} & c_{13}^{E} & 0 & 0 & 0\\ c_{13}^{E} & c_{13}^{E} & c_{33}^{E} & 0 & 0 & 0\\ 0 & 0 & 0 & c_{44}^{E} & 0 & 0\\ 0 & 0 & 0 & 0 & c_{44}^{E} & 0\\ 0 & 0 & 0 & 0 & 0 & {\frac{\left(c_{11}^{E}-c_{12}^{E}\right)}{2}}_{} \end{array}\right]e_{ij}=\left[\begin{array}{cccccc} 0 & 0 & 0 & 0 & e_{15} & 0\\ 0 & 0 & 0 & e_{15} & 0 & 0\\ e_{31}{}_{} & e_{31} & e_{33} & 0 & 0 & 0 \end{array}\right]\varepsilon_{ij}^{S}=\left[\begin{array}{ccc} \varepsilon_{11}^{S} & 0 & 0\\ 0 & \varepsilon_{11}^{S} & 0\\ 0 & 0 & \varepsilon_{33}^{S}{}_{} \end{array}\right]$$

The determination of values for the 10 parameters in the linear piezoelectric constitutive equations can be addressed by different techniques. A classical method is presented in the IEEE standard [3] where different samples are needed. In this standard, different samples must be cut and polarized to enlarge specific resonant modes. The objective is to decouple resonance modes to simplify the determination of the parameters. However, this procedure is laborious. It uses different samples that all have small differences between them. The piezoceramic is not the same from one sample to another; this is intrinsic to all manufacturing procedures. This type of test may result in imprecise identification of the parameters for practical purposes. As an alternative, full identification of the model parameters using finite element method (FEM) simulations is a successful tool for solving this problem [5,6]. The objective is the same, to determine the parameters in the model and there are plenty of different strategies that can be used to implement this iterative procedure [6,7,8].

One of the proposed strategies divides the parameters into groups to simplify the solution [9,10]. On one side, it is recognized that the real part of the model is responsible for the frequency of the resonant modes and the imaginary part is associated with the energy losses. Then the problem is divided in two. Firstly, the real part of the model is solved, and then once that solution is good enough, the imaginary part is considered. On the other side, acknowledging that the problem has higher sensitivity to some parameters, the model is fitted for the more sensitive parameters first.

When the solution is close for the more sensitive parameters, many optimization algorithms can be used. As an example, we used the classic Nelder–Mead algorithm [11].

In this work, we propose the use of neural networks (NN) to identify the parameters of the constitutive equations shown in Equation (1). The obtention of these parameters allows the precise simulation of piezoelectric materials using FEM simulations. The main advantage of the NN approach is its extremely fast computation for a new sample (once the model is trained). There are several fast techniques to characterize materials using iterative methods and gradient optimization [12]. For example, our research group developed a very fast version using the moving asymptotes method to implement a gradient-based optimization algorithm for piezoelectric materials [13]. However, all iterative methods are several orders of magnitude slower than the NN computation. In the NN approach, only the evaluation of functions are performed whereas many FEM simulations are needed in the iterative methods.

Here we reproduce the strategy adopted in [9], implementing the optimization only for the more sensitive parameters. This strategy divides the problem into two steps, one to determinate the more sensitive parameters and the second is a final refinement to adjust all parameters in the model. The first step is the more important because it adjusts the most important parameters for most applications. This strategy is commonly used today for the precise determination of the model. The use of the NN provides a new approach to perform this first step, and maybe various other NN implementations might solve the problem more completely. This is a first proof of concept using this approach, and we demonstrated that this technique may be an efficient way to solve the problem. 

The use of NN to identify material properties has been reported by several researchers [14,15,16]. In the case of piezoelectric materials, the use of NN is reported to identify nonlinearities as hysteresis and the behavior close to the resonance of a flexural actuator [17,18,19]. However, we have no references regarding the use of this technique to solve the problem of identifying the parameters in the piezoelectric model.

## 2. Forward Optimization vs. Neural Network Approach

In recent years, the identification of the piezoelectric model using FEM optimization has gained popularity. In this section, we highlight the main differences between the classical methods and the neural network approach. 

**Figure 1:**
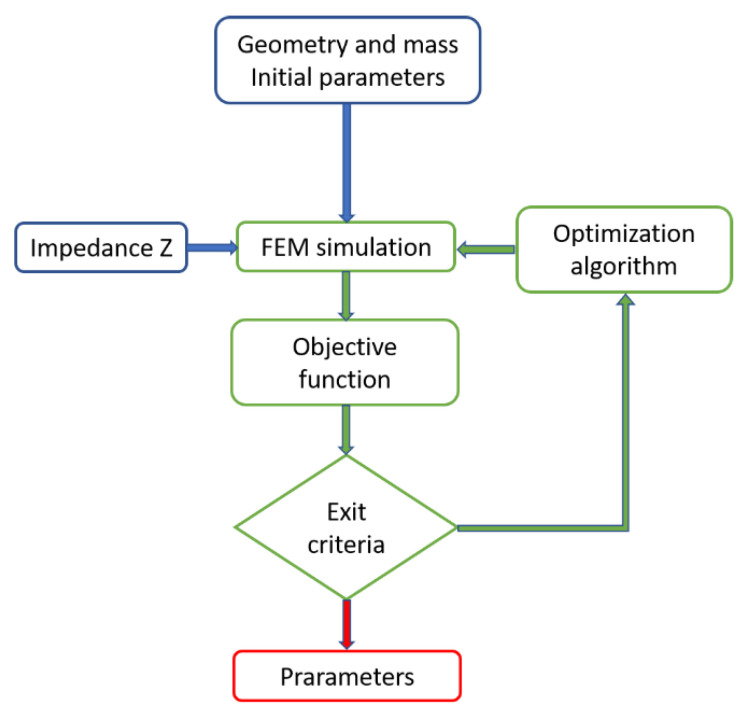
Flow chart of the forward optimization approach using Finite Element Method (FEM). Blue indicates the input data, green the calculus performed at each step in the loop and red the results.

Classical methods start from an initial seed and then a first simulation is performed. In order to reproduce the impedance curve from FEM simulations, the constitutive parameters set (c_11_, c_12_, c_13_, c_33_, c_44_, e_31_, e_15_, e_33_, ε_11_, ε_33_) (see Equation (1)) is needed, and the mass density and geometric information must also be known. Using this information, the FEM simulation allows the electrical impedance response and the mechanical displacement in the nodal points to be obtained. Thus, this is called forward optimization, the computation is a simulation from the parameter space to the impedance response space. Figure 1 shows the flow chart for the forward optimization approach.

Here we can distinguish several ingredients or decisions to be made that affect the final solution: a start point or initial seed, the computation of a FEM algorithm, an objective function to measure the difference between the simulated and the experimental data, an exit criterion, and an optimization algorithm. The main drawback of this technique is the computation time involved in the FEM simulation. An iterative loop of FEM simulations must be performed for each sample to be identified.

**Figure 2:**
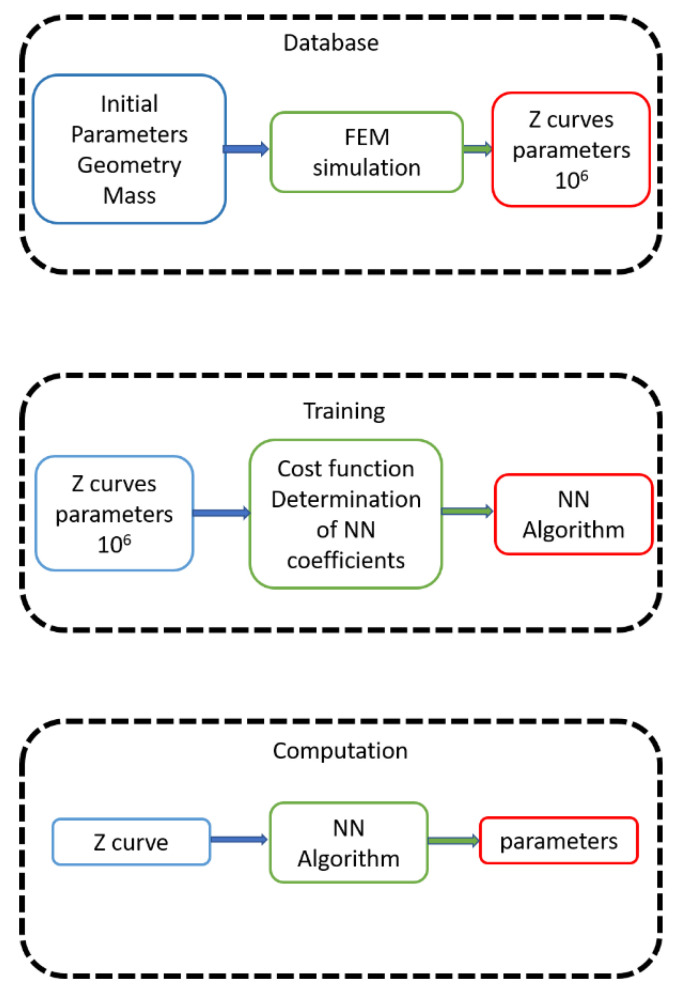
Neural network (NN) approach. Blue indicates the input data, green the calculus and red the results of each phase. Each phase has an input, computation, and a result.

On the other hand, we have the neural network approach. This includes three very different phases: the construction of the database, the training phase, and the computation phase. To construct the database, many FEM simulations are performed from an initial seed. The simulations are similar to those used in the forward approach. Each parameter in the model is changed from an initial condition, generating a large family of impedance curves linked to parameters sets. In the training phase, the network is trained using the database impedance curves simulated by FEM. Using this information, the NN constructs the function that predicts a parameter set starting from the impedance curve. An objective function, an exit criterion and an optimization algorithm are necessary to train the NN. Then, in the computation phase, the NN is ready to use, and it quickly solves the problem using the impedance curve as input and it gives the parameters as output. Figure 2 shows the flowchart of this approach.

At the end of both processes, we obtain a set of parameters. This set can be used to simulate the sample, and thus, a measure of the difference between the original data and the numerical result can be obtained. Now we highlight the main issues in both techniques in order to understand the advantages in both cases. 

### 2.1. Initial Seed 

Both techniques need a start point for the parameters. In the forward approach, this is the first attempt at running the iterative process. In the neural network, the database is constructed around the starting point. 

### 2.2. FEM Simulation 

Both techniques require the realization of FEM simulations. In the forward approach, one simulation is performed at each step of the iterative process, and depending on the convergence a few hundred simulations are needed. In the neural network, the FEM simulations are generated in the construction of the database. In the example presented in this paper we performed about a million simulations. Of course, this is very time consuming, but it is only done to construct the database. The database can be used to train several architectures of NN. After training the NN, the algorithm to evaluate the parameters is very fast compared to the loop of the forward approach. 

### 2.3. Optimization Algorithm

Both techniques have an algorithm to determine the parameters. In the forward approach, this is the set of rules or the computation to determine the next parameter set in the loop. This computation is performed in all steps of the optimization loop. On the other hand, for the NN, the optimization is done during the training phase, but later, for each new sample the fitting algorithm runs only one time, providing the output without iterations. 

### 2.4. Objective Function 

The objective function is the one to be minimized during the optimization process. Here, we have another big difference between both techniques. The forward approach minimizes the difference in the impedance curve (or some computation related to the impedance), and this difference is evaluated step-by-step in the optimization iteration. The neural network approach constructs the algorithm by training the network using simulated curves. In these simulated curves the set of parameters is known, and the objective function is computed in the parameter space. 

### 2.5. Exit Criteria

In the forward approach the exit criteria must be evaluated at each step in the loop whereas in the neural network the criteria are given in the training phase to ensure the convergence of the results. 

## 3. Materials and Methods

In this section, we discuss the main ideas regarding the proposed solution and the strategy used in the implementation. This work may be interesting for piezoelectric material manufacturers and also end users that work with a fixed geometry and material. Technical details about the implementation of the algorithms are detailed in the MSc. thesis [20] of M. del Castillo. 

The general strategy was to implement a neural network to determine the more sensitive parameters, $\left[c_{11},c_{13},c_{33},c_{44},e_{33}\right]$. The sensitivity analysis to determine this set is presented by Pérez et al. [5,9,10] for both the real and the imaginary part. This sensitivity analysis is valid for discs where the diameter is much greater than the thickness. The permittivity $\varepsilon_{33}$ is also a sensitive parameter, however as it can be estimated directly from the impedance curve, following the guidelines presented in the IEEE standard [3]; it was not included in the chosen set.

The methodology was implemented for a specific sample, which is described in Section 2.1. The neural network was trained using FEM simulations, Section 2.2 describes the FEM implementation and Section 2.3 provides a detailed description of the neural network.

### 3.1. Selected Sample

In order to implement the proposed technique, we selected a synthetic sample, the commercial PZ27 from Meggitt (Ferropem Piezoceramics, Kvistgaard, Denmark) [21] with a diameter of 20 mm and thickness of 2 mm and a mass density of 4.85 × 10^6^ kg/m^3^. Table 1 shows the real constitutive parameters obtained from [10].

This sample was used as the base to train the network. Each parameter was changed by 10% around the initial value to provide the complete set of impedance curves. To evaluate the dispersion of the parameters in PZT27 we compared them with the results obtained in [22] for a family of 60 samples with 1 mm and 2 mm thickness, and 10 mm, 20 mm and 30 mm diameter (ten samples of each size). The results showed a dispersion of less than 3% in the worst case.

### 3.2. Finite Elements

FEM software implemented in MATLAB (R2018b 1994–2021 The MathWorks, Inc.) was used to perform the simulations. It is important to note that every FEM code that allows the asymmetric simulation of piezoelectric elements can be used for this application. The use of a bidimensional symmetry is mandatory for the time involved in the simulations, and the use of 3D elements is not possible given the actual computing capacity. 

Elements with a square shape were selected. A previous convergence analysis obtained good results using 30 elements in the thickness. Here, we made a trade-off between the simulation time and memory use with the convergence of the results. 

Also, the FEM methods must have a mechanism to introduce the energy losses, for example Rayleigh damping parameters [23,24] or complex parameters. In this work, we selected the use of complex parameters, but the complex part was kept fixed. More details about the implementation of the finite element code can be found in the work of Perez et al. [10]

### 3.3. Neural Network Implementation

Here, we present the main characteristics of the neural network implemented for this work. The neural network optimization technique performs all the FEM simulations at an early stage, it uses the simulations to train the NN, and then, the outcome of the process is a complex function that given the impedance curve, returns the proposed parameters.

**Figure 3:**
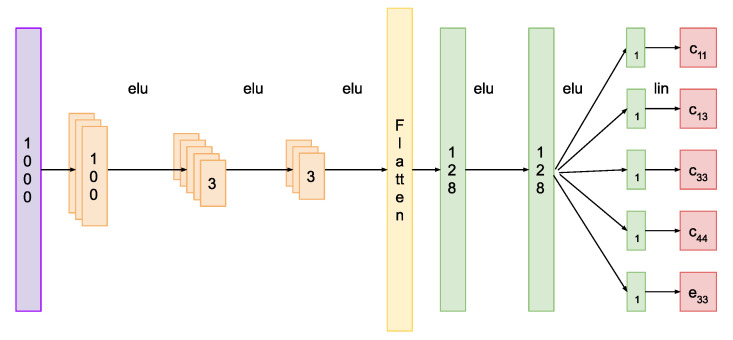
Architecture of the proposed Neural Network.

The chosen architecture for the implemented NN was a 1-dimensional convolutional architecture [25,26]. It takes the full impedance curve vector (1000 data points) as input and it outputs five values corresponding to the sensitive selected set, as mentioned earlier. It is composed of three convolutional layers. Each layer consists of a group of convolution operators and a non-linear activation function. The network then has a reshaping layer that allows the interconnection with the final stage. Then, we have two fully connected layers, each one with its non-linear activation. Finally, there are five single neurons, each followed by a linear activation; these are the outputs of the network. The non-linear activation of choice, in this case, was the exponential linear unit all through the network, but others might also work as well. Furthermore, no batch normalization was used for the network, and no pooling layers were included. Figure 3 shows a schematic representation of the network architecture, the numbers in the boxes represent the kernel dimension for the convolution filters of each layer.

Training a NN is an optimization process where, given a proposed model and some data, the objective is to find the parameters of the model that better fit the data. Thus, there is a need to define an objective function or error in the parameter space for this approach.

As an error function for this process, a linear combination of the mean square error of each parameter was used. Furthermore, an absolute mean percentage error was used as a metric to monitor the progress of the training phase, and to evaluate the final performance of the network.

For all computations, the parameters were normalized by the values of Table 1. This was done following Equation (3), where $p^{k}$ is the database parameter, $p_{\mathrm{FEM}}^{k}$ is the real value of the parameter that generated the curve and $p_{\mathrm{seed}}^{k}$ is the value of the parameter in Table 1.

(3) $$p^{k}=p_{\mathrm{FEM}}^{k}/p_{\mathrm{seed}}^{k}$$

Then, they all have the same weight and are close to one. Nevertheless, the progress was independently monitored for each parameter using:

(4) $$MAPE_{P}=100\frac{1}{N}\overset{N}{\underset{k=1}{\sum }}\frac{\left|P^{k}-P^{k}{}_{NN}\right|}{\left|P^{k}\right|}$$

where $P^{k}$ are the parameters of the database for each simulated curve, and $P{\;}^{k}{}_{NN}$ are the prediction of the NN for the same simulation; this computation is averaged over the training and validation set. The network was trained with simulated data. As a starting point for the data generation, a real ceramic was chosen, and the selected parameters were randomly disturbed in a 10% range from the original value to generate the dataset. With 600,000 impedance curves and their corresponding parameters the network was trained during 220 epochs, and it was later tested in a 100,000 set.

### 3.4. Database Creation

The biggest advantage of using the NN approach is that after the network is trained, no more FEM simulations are needed to determine the parameters. All simulations are used in the training of the NN and in the evaluation of the performance. In this work, the simulations were: 600,000 for training, 100,000 for validation during the training, 100,000 to evaluate the results in the subspace (c_11_, c_13_, c_33_, c_44_, e_33_) and another 100,000 to evaluate the performance in the full parameters’ space.

**Figure 4:**
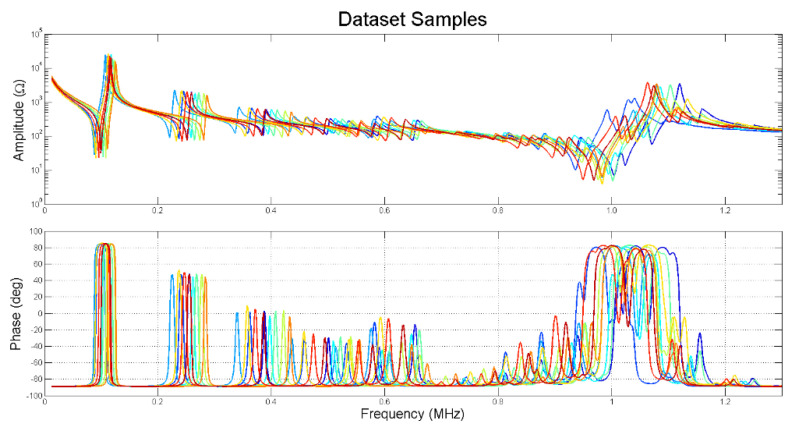
Impedance curves for 10 samples randomly picked in the dataset.

The size of the database is related to the number of parameters to be identified and range of variation of the parameters. In this first work, we identified the five most significative parameters, and the range of variation was ±10% around the initial point. This database is suitable for this piezoceramic with this geometry. The values to generate the curves were chosen at random using a flat distribution. In Figure 4, ten different samples from the dataset are plotted to show the variations in the impedance curve space. 

## 4. Results

In this section, we present two different sets of results. The first are for the solution of the problem in the restricted sub space were the NN was trained. The network was trained for the more sensitive parameters (c_11_, c_13_, c_33_, c_44_, e_33_) while the others remained fixed $\left(c_{12},e_{31},e_{15},\varepsilon_{11},\varepsilon_{33}\right)$. In the first case, the results were evaluated using samples of the same subspace. 

Next, we evaluated a more realistic situation, where the results were evaluated using the full set of parameters chosen at random in a 10% range. 

### 4.1. Results in the Restricted Subspace

During training, the evolution of the error variables was very slow. However, there were improvements in all steps, both in training and validation. The network was trained using 600,000 synthetic impedance curves, another 100,000 were used for validation during training and 100,000 to evaluate the results later (Test set). The selected model has only five outputs, the subset (c_11_, c_13_, c_33_, c_44_, e_33_). In this case, it was possible to follow each constitutive parameter over the training stage. Table 2 shows the mean values obtained for the validation set and the test set.

Results need to be evaluated in a set of data never seen by the NN to ensure there is no overfitting to the data used during training. In this case, the table above shows that this phenomenon did not occur here.

**Figure 5:**
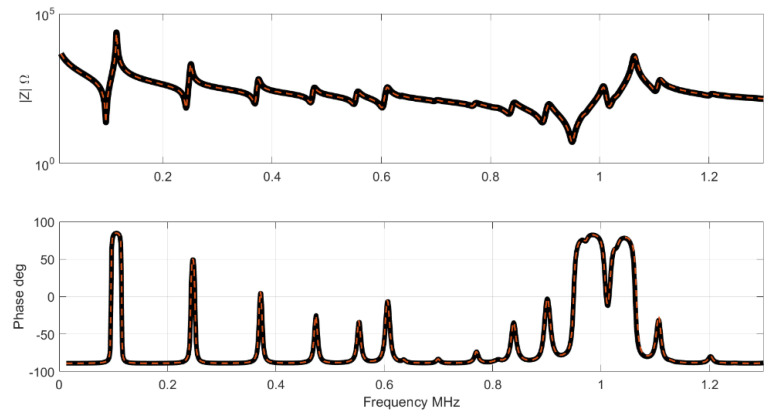
Impedance results. The black curve is the impedance of the Test set, the red curve is the NN result.

**Figure 6:**
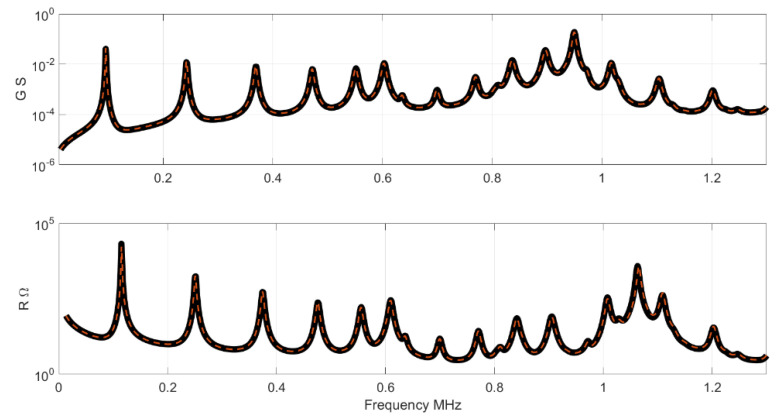
Results for conductance and resistance. The black curve is the impedance of the Test set, the red curve is the NN result.

In order to show the results in the impedance curve, one sample randomly selected from the Test set was plotted. Figure 5 shows the impedance, modulus and phase of the selected Test sample and the result given by the NN. Figure 6 shows the results for the resistance R and conductance G. The G and R peaks highlight the resonance and the antiresonance of the sample. Note that the NN results are parameters, to obtain the impedance, a FEM simulation must be performed after the computation. FEM simulation requires a full set of parameters to produce an impedance curve. Thus, the less sensible parameters used for these simulations are those in Table 1.

We can define a mean quadratic error to evaluate the difference between both curves, the Test and that obtained by *NN*. The error in ppm is defined as

(5) $$err_{Z}=10^{6}\frac{\sum_{\omega_{1}}^{\omega_{N}}{\left(\left|Z_{Test}\left(\omega_{i}\right)\right|-\left|Z_{NN}\left(\omega_{i}\right)\right|\right)}^{2}}{\sum_{\omega_{1}}^{\omega_{N}}{\left|Z_{Test}\left(\omega_{i}\right)\right|}^{2}}$$

For a selected subset of curves, the error computed in Equation (5) has a mean value of 237 ppm and a maximum of 804 ppm. In the parameter space, the error between the Test samples and the results of the *NN* can be defined as

(6) $$err_{P}=10^{6}\frac{{\left(P_{Test}-P_{NN}\right)}^{2}}{P_{Test}^{2}}$$

Here *P* is one element of the subset of parameters (c_11_, c_13_, c_33_, c_44_, e_33_) and the results are in ppm. Table 3 shows the mean and the maximum $err_{P}$ for each parameter. 

### 4.2. Results with the Full Set of Parameters

To evaluate the robustness of the NN, the network was tested again using a different Test set with the full set of parameters changing at random. This is a more realistic situation. In practice we do not know the real value of any of the parameters and all must be identified. The new Test set also has a size of 100,000. Table 4 shows the results obtained in this case. 

For a newly selected subset of curves, the error computed in Equation (4) has a mean value of 1044 ppm and a maximum of 2329 ppm. This is a statistical result for the Test set. In a practical application, this is more than enough to simulate the behavior of the sample.

**Figure 7:**
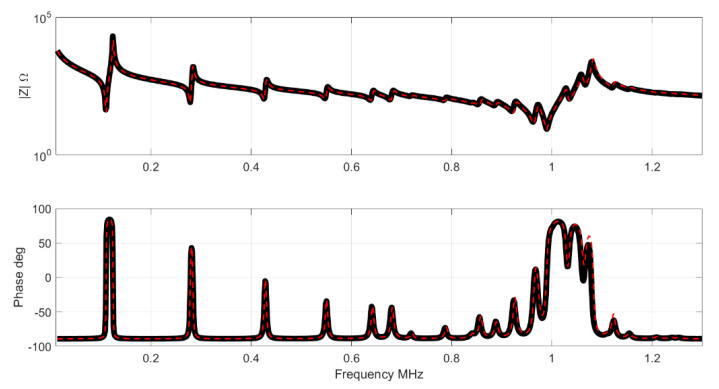
Results Impedance for the full set. The black curve is the impedance of the Test set, the red curve is the NN result.

**Figure 8:**
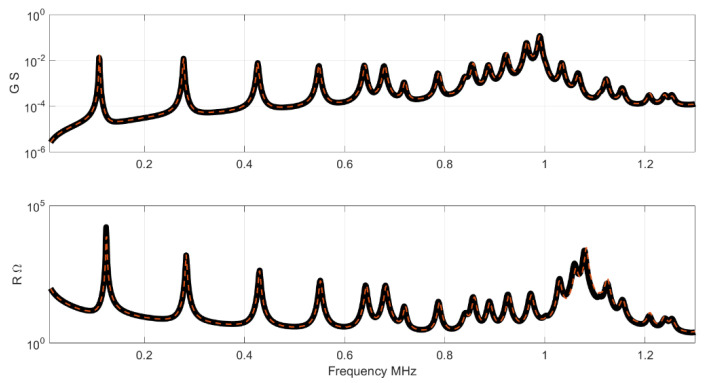
Results of conductance and resistance for the full set. The black curve is the impedance of the Test set, the red curve is the NN result.

In this case the differences between both curves can be clearly observed. Figure 7 and Figure 8 shows the impedance and the G-R representation in this case. Note the differences close to the antiresonance of the thickness mode.

## 5. Discussion and Conclusions

This work is a first proof of concept in the use of neural networks for the identification of the parameters in a linear piezoelectric model. In this first approach, we proved the ability of the NN to determinate the more sensitive parameters. In the analysis of an impedance curve to determine the parameters of the constitutive equations, the determination of the sensitive parameters is very demanding. This determination requires a fast and robust method. Once the sensitive parameters are close to the solution, there are several efficient techniques that can be used to obtain the final solution, for example, the Nelder–Mead nonlinear minimization. 

To train the network, a first seed for the parameters that is close to the solution is necessary. In the training phase, hundreds of thousands of curves are simulated by FEM. However, after the NN is ready, computation is extremely fast without the need to perform new FEM’s simulations. 

Results were tested using two different sets of curves, the first restricted to the subspace of the more sensitive parameters and the other with all parameters changing at random. The error in the restricted subspace is one order less, however, in the full problem the agreement is still suitable to be used in a later Nelder–Mead minimization.

The main difficulty of the proposed approach is the need for a first approximate solution. In the case of a new material or a material without a previous numerical characterization of the full set of parameters, we cannot apply this methodology. However, the methodology could be very useful for a manufacturer of piezoceramics. Manufacturers work with the same material and a limited family of geometries. Thus, the first characterization can be performed by another methodology, and after that a NN could be trained for each geometry. In this case, we can obtain the properties for each fabricated sample, without delay and without a specialist, to characterize the production. This first contribution shows the feasibility of the technique to solve the problem. Next steps include the generalization for the full parameter set and testing the application with real piezoceramics. 

## Figures and Tables

**Table 1:** PZ27 real parameters.

C_11_	C_12_	C_13_	C_33_	C_44_	E_31_	E_15_	E_33_	ε_11_/ε_0_	ε_33_/ε_0_
118.1	74.9	73.8	110.4	20.3	–5.1	11.2	16.0	984	830

Units: (C) in GPa and (E) in N/m^2^.

**Table 2:** Average evaluation of the results in the parameter subspace.

	C_11_	C_13_	C_33_	C_44_	E_33_
**MAPE_val_**	0.48	0.43	0.31	0.57	0.59
**MAPE_test_**	0.48	0.43	0.32	0.57	0.59

**Table 3:** Average evaluation of the results in the parameter space in ppm.

	C_11_	C_13_	C_33_	C_44_	E_33_
**Err_P_ (Aver)**	3.5	2.9	3.3	4.5	6.6
**Err_P_ (Max)**	15	5.8	5.1	11.7	21.4

**Table 4:** Average evaluation of the results in the full parameter space.

	C_11_	C_13_	C_33_	C_44_	E_33_
**MAPE_test_**	1.3	1.3	0.38	0.83	2.6

## Data Availability

The data presented in this study are available on request from the corresponding author.
